# Programming of Cardiovascular Dysfunction by Postnatal Overfeeding in Rodents

**DOI:** 10.3390/ijms21249427

**Published:** 2020-12-11

**Authors:** Marie Josse, Eve Rigal, Nathalie Rosenblatt-Velin, Luc Rochette, Marianne Zeller, Charles Guenancia, Catherine Vergely

**Affiliations:** 1 Research Team of Physiopathologie et Epidémiologie Cérébro-Cardiovasculaires (PEC2, EA 7460), Faculté des Sciences de Santé, Université de Bourgogne-Franche Comté, 21000 Dijon, France; Marie_Josse01@etu.u-bourgogne.fr (M.J.); eve.rigal@u-bourgogne.fr (E.R.); luc.rochette@u-bourgogne.fr (L.R.); marianne.zeller@u-bourgogne.fr (M.Z.); charles.guenancia@chu-dijon.fr (C.G.); 2 Division of Angiology, Heart and Vessel Department, Centre Hospitalier Universitaire Vaudois and University of Lausanne, 1005 Lausanne, Switzerland; nathalie.rosenblatt@chuv.ch

**Keywords:** perinatal programming, postnatal overfeeding, rodents, heart, arteries, ischemia, cardiovascular dysfunction

## Abstract

Nutritional environment in the perinatal period has a great influence on health and diseases in adulthood. In rodents, litter size reduction reproduces the effects of postnatal overnutrition in infants and reveals that postnatal overfeeding (PNOF) not only permanently increases body weight but also affects the cardiovascular function in the short- and long-term. In addition to increased adiposity, the metabolic status of PNOF rodents is altered, with increased plasma insulin and leptin levels, associated with resistance to these hormones, changed profiles and levels of circulating lipids. PNOF animals present elevated arterial blood pressure with altered vascular responsiveness to vasoactive substances. The hearts of overfed rodents exhibit hypertrophy and elevated collagen content. PNOF also induces a disturbance of cardiac mitochondrial respiration and produces an imbalance between oxidants and antioxidants. A modification of the expression of crucial genes and epigenetic alterations is reported in hearts of PNOF animals. In vivo, a decreased ventricular contractile function is observed during adulthood in PNOF hearts. All these alterations ultimately lead to an increased sensitivity to cardiac pathologic challenges such as ischemia-reperfusion injury. Nevertheless, caloric restriction and physical exercise were shown to improve PNOF-induced cardiac dysfunction and metabolic abnormalities, drawing a path to the potential therapeutic correction of early nutritional programming.

## 1. Introduction

Early growth trajectories [1] are essential to determine maturity metabolism and health in adulthood. Foetal exposure to toxic components, endocrine disruptors and to under- or over-nutrition are key determinants in the development of metabolic diseases later in life and are all related to the concept of developmental programming [2]. This theory suggests that hormonal or nutritional factors in the neonatal period can lead to major changes in gene expression, initially intended to adapt the organism to changes in its environment. However, these changes may also be inappropriate and predispose the individual to develop organ malfunction and diseases if the postnatal environment does not match with the orientation of genomic expression [3].

A recent study reported that, during the period 2006–2016, 17.9% of European children aged 2–7 were overweight or obese [4]. As it is now perfectly recognized, obesity and being overweight are predisposing factors for metabolic and cardiovascular diseases, and it is therefore essential to pay great attention to the consequences of unsuitable diet during childhood and to overnutrition, which may induce an increased risk of developing non-communicable diseases and late onset metabolic disorders [5].

In rodents, postnatal overfeeding (PNOF), induced by litter size reduction after birth, has been shown to induce a significant weight gain at weaning (30% increase) that remains in adult life, although at a lower level (15%), and is associated with increased adiposity and altered metabolic parameters [6]. Indeed, litter size reduction leads to increased availability of breast milk for pups [7,8], and some studies have also shown that milk from small litter dams was high in energetic value with an enrichment in triglycerides [7,9] but not proteins [10], in comparison to control dams. Thus, PNOF represents a relevant experimental model to study the long-term impact of overnutrition in the early stages of life [11,12]. In fact, in most mammals, the development of some organs is not completed at birth and continues in the immediate neonatal life. In contrast, the heart was for a long time considered to be a post-mitotic organ with a definite number of cardiomyocytes at birth, which were believed to be unable to proliferate or regenerate. This observation was challenged when studies demonstrated, in humans and animals, that a low regeneration rate of cardiomyocytes was still possible [13,14,15,16,17]. Thus, stimuli induced by neonatal malnutrition may disturb cell proliferation and/or differentiation, and affect, in the short- or long-term, the heart and vascular function.

**Figure 1:**
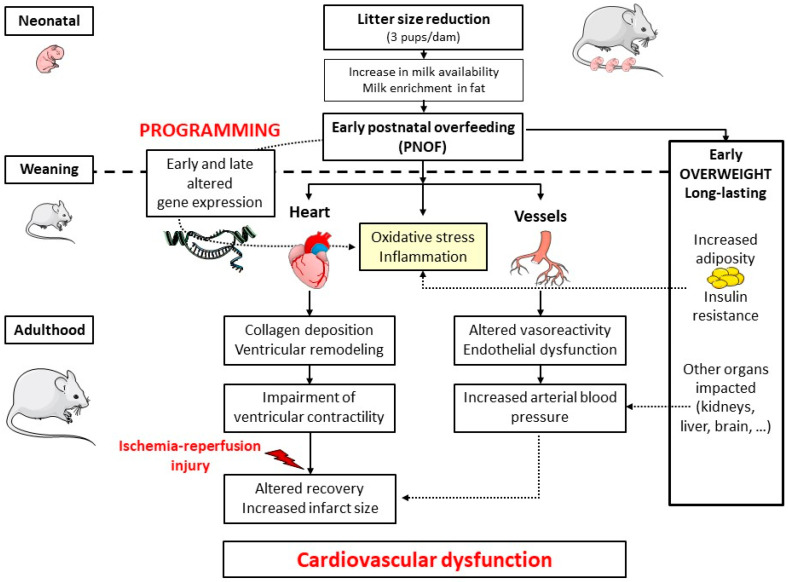
Cardiac and vascular consequences of neonatal programming by postnatal overfeeding (PNOF) in rodents. PNOF can be induced by litter size reduction, a situation that increases milk consumption during the 24 days after birth and leads to early and long-lasting obesity. Additionally, the expression of several genes is modified in many organs, leading to structural and metabolic alterations. Cardiovascular consequences appear later in life, including altered vasoreactivity and increased arterial blood pressure, ventricular remodelling, cardiac oxidative stress and inflammation, and impaired cardiac contractility and architecture. Altogether, these modifications are responsible for an increased sensitivity to ischemia-reperfusion injury and cardiovascular dysfunction in adulthood.

In this review, we explore how PNOF impacts the cardiovascular system at early life stages and later in adulthood, and the potential underlying mechanisms (Figure 1). All data presented in the article are summarized in Table 1.

## 2. PNOF is Associated with Systemic and Cardiovascular Metabolic Alterations

As observed earlier by Plagemann [39,40,41,42], rodents subjected to PNOF have a significantly higher body weight than controls at weaning, which persists through growth and maturation. This permanent increase in body weight is not related to a general enhancement of body growth including bones, muscles and organs, but rather to increased adiposity [43]. Whole body composition analysis with magnetic resonance imaging (MRI) demonstrated that PNOF mice have more body fat mass and less lean mass [32]. Deeper analyses of fat location showed that PNOF rats have an increase in both subcutaneous and visceral fat (epididymal, retroperitoneal, perirenal, mesenteric) at weaning [20] and later [7,44,45], with an increased adipocyte surface [46,47,48]. The white adipose tissue of PNOF rodents also displays significant differences in the expression of proteins involved in insulin responsiveness, such as a downregulation of insulin receptor substrate-1, protein kinase B (Akt2) and glucose transporter 4 [49] and in the adipose glucocorticoid metabolism [46,47,50,51]. An overexpression of proinflammatory cytokines, such as tumour necrosis factor (TNF)-α, TNF-receptor 1, interleukin-6 and resistin, is also observed in the white adipose tissue of PNOF rats [50]. Recent data also suggest that PNOF may impair the differentiation potential of subcutaneous adipose mesenchymal stem cells, which may be involved in the increased adiposity observed in neonatally overfed mice [52]. This disequilibrium of fat proportion is associated with other endocrine and metabolic changes. A significant increase in insulin plasma levels was reported in overfed animals, in both rats and mice, young and adult [6,22,32,37,46,47,53]. Such an increase is believed to reflect insulin resistance [12], an hypothesis corroborated by a higher fasting glucose level in overfed animals [6,22] but also impaired glucose tolerance [7,9,22,26,54] and increased insulin secretion in response to glucose [7,39]. Plasma leptin levels were also shown to be higher in PNOF animals [7,21,22,37,46,55], an observation that could easily be explained by increased adiposity, since leptin production is released from adipocytes and the gene expression of leptin is increased in the white and brown adipose tissue of PNOF rats [56]. When adiposity is increased, circulating lipid levels are also disturbed in PNOF rodents. Significant increases in free fatty acids, triglycerides and cholesterol content associated with decreased HDL-cholesterol levels were described in most studies [21,23,25,29,46,49], while some others did not detect any lipid level variations [6]. These discrepancies may be related to distinct strains, ages and pathophysiological conditions when lipid profiles were analysed. The modification of the lipid profile and adipose tissue content may be responsible for a pro-inflammatory state; indeed, adipose tissue is able to release adipocytokines and some of them have been described to be involved in the development of cardiovascular diseases [57,58].

Analyses of cardiovascular metabolism also demonstrated an alteration of insulin signalling in the myocardium of overfed rats [21] and mice, confirmed by decreased insulin receptor (IR)-β and IR substrate-1 (IRS) phosphorylation [36]. These impairments appear very early, since they are present at weaning in PNOF mice hearts with decreased expression of IRβ, pTyr-IRS1, phosphatidyl inositol-3 kinase (PI3K), type-4 glucose transporters (GLUT4) and pAkt/Akt, and increased expression of GLUT1 and pAMPKα/AMPKα content [38]. The immunofluorescence of glucose transporter 4 (GLUT-4) location in cardiomyocytes also showed a decreased translocation of GLUT-4 to the plasma membrane [21]. However, another study observed that GLUT-4 protein expression was increased in overfed rats [22]. Indeed, experiments on isolated aorta and hearts showed decreased responsiveness to insulin due to under-activation of the PI3K/Akt pathway and over-activation of the MAPK cascade, respectively [53]. In both myocardium and arteries, insulin resistance may be related to a pro-inflammatory state, since levels of pro-inflammatory cytokines and markers of a pro-oxidant state were significantly increased [21].

In the juvenile hearts of post-weaning PNOF mice, the activities of lactate dehydrogenase and citrate synthase were increased, accompanied by enhanced carbohydrate oxidation, lower mRNA expression of carnitine palmitoyl-transferase I (CPT1) and peroxisome proliferator-activated receptor-α (PPARα), and increased uncoupling protein-2 (UCP2) expression, indicating a switch in cardiac metabolism preference towards carbohydrates [38].

## 3. PNOF Affects Blood Pressure and Vascular Function

In vivo measurements of blood pressure revealed a moderate but significant 10–20 mmHg increase in both systolic and diastolic blood pressure in adult male rats and mice [11,19,24,29,31,56,59]. Blood pressure is highly dependent on cardiac flow and vascular resistance. Analyses of vascular reactivity in arteries collected from PNOF rodents showed differing results. On isolated aortic rings of adult overfed rats, some authors demonstrated a decreased vasorelaxation in response to acetylcholine (Ach) but not to sodium nitroprusside (SNP) [29], which could suggest impaired endothelial function [60]. Insulin exerts an endothelial-dependent vasodilatory effect through increased nitric oxide (NO) production and availability in endothelial cells [61]. Experiments on vascular reactivity pointed out that overfed rats have a decreased vasodilatory capacity in response to insulin due to a reduced phosphorylation and activation of the enzyme eNOS involved in NO production [21]. The same other authors showed an impaired response to both Ach and SNP [21], meaning that both endothelial and smooth muscle cell responses were altered. Additionally, contractile response to phenylephrine was also shown to be increased in aortic segments from overfed rats [29], suggesting an enhanced response to α-adrenergic-dependent vasoconstrictive stimuli. However, we failed to detect differences in vascular reactivity between control and overfed animals [6]. Altogether, these results indicate that vascular reactivity can be altered by PNOF, but might be influenced by strains, breeding and the maturity stages of animal models.

In addition, vascular morphological alterations can be observed in adult overfed rats, with increased collagen content and lower elastic fibre density [27], aortic wall thickening in parallel with reduced elastin integrity and increased blood pressure [24]. Higher aortic and plasma expression and activity of matrix metalloproteinase-2 (MMP-2) were evidenced, and the aortic MMP-2 activity was also shown to be positively correlated with aortic wall thickness [24]. These authors suggest that aortic MMP-2 upregulation could be explained by metabolic disorders such as elevated plasma insulin and/or lipid levels, considering that such disorders are usually observed in overfed animals as indicated previously [62].

Other parameters involved in blood pressure regulation may also be affected by PNOF. For instance, impaired renal function is also a strong determinant in the development of cardiovascular diseases. Many studies reported that PNOF rats developed systemic hypertension associated with renal dysfunction in adulthood [18,63]. Impaired kidney function and structure were observed in both young [28] and adult [18,35,64] overfed rats, with an initial increased glomerular endowment, followed by progressive glomerulosclerosis, accelerated reduction in nephron number, cortical cell apoptosis [19], altered glomerular filtration rate [18] and higher senescence in kidneys with aging [35]. Moreover, the intra-renal renin angiotensin system is disturbed, with an increased expression of renin and angiotensin II type (AT) 2 receptor in overfed rats [59,65]. Considering the major importance of the renin angiotensin aldosterone system in regulating blood pressure, these modifications may have an impact on arterial blood pressure levels in PNOF rodents.

Additionally, some authors have also shown that PNOF was able to increase the capacity of the adrenal gland to secrete catecholamines [66] but also corticosterone [46,47]. Regarding the pro-hypertensive effects of these adrenal hormones, a humoral dysregulation could also be involved in PNOF-driven hypertension.

Thus, not only altered vascular reactivity and structure, but also PNOF-induced renal and humoral dysfunction might play a role in the development of increased arterial blood pressure.

## 4. PNOF Affects Heart Structure, Function, and Adaptation to Pathological Situations

### 4.1. PNOF Impairs the Architecture and Structure of the Heart

Metabolic alterations induced by PNOF may also change the structure of the heart by modulating cardiac cell fate. In fact, PNOF occurs at a critical stage of heart formation. Indeed, at birth, mammalian hearts switch from a low oxygen environment to 20% oxygen. This leads to a change of metabolism from anaerobic glycolysis to oxygen-dependent mitochondrial oxidative phosphorylation [67]. This metabolism switch is required for body and organ growth. Concomitantly, hearts progressively lose their capacity to regenerate. Cardiomyocytes exit from the cell cycle, stop their proliferation and undergo hypertrophy and architecture maturation [68]. Fibroblasts become larger, with a reduced cell turnover compared to foetal fibroblasts [69]. Furthermore, after birth the composition and stiffness of the extracellular matrix (ECM) change. Indeed, adult heart ECM is enriched in collagen 1 and laminin, with reduced levels of fibronectin and periostin [70]. Altogether, these results suggest that metabolic changes will directly affect the cardiac cells and thus the heart structure. This was reported in several overfeeding animal models such as zebrafish, rodents and sheep [29,30,71,72]. Numerous studies reported impaired cardiac morphometry and hypertrophic protein marker expression.

At the organ level, PNOF, which is associated with cardiac hypertrophy at weaning and/or in adults, is characterized by an increase in cardiomyocytes’ diameter or in heart mass normalized to tibia length [23,29,36,37]. Moreover, PNOF rat hearts have left ventricular hypertrophy with an increased area of cardiomyocytes and a decreased vessel density [23]. However, myocardial hypertrophy is not observed in all studies [6,32,56]. Metabolic changes may directly modulate cardiomyocyte fate, and important cellular mechanisms will be impaired depending on the stage of heart development, such as cardiomyocyte cell proliferation, maturation, or survival. For example, during development, glycolytic metabolism was shown to be associated with cardiomyocyte cell proliferation [8], and glucose inhibits cardiomyocyte maturation [8,73]. In the same line, the switch from carbohydrates to fatty acid metabolism increases cell maturation and blocks cardiomyocyte proliferation [74]. In contrast, inhibiting fatty acid metabolism stimulates cardiomyocyte proliferation in postnatal hearts, which leads to the extension of the postnatal cardiomyocyte proliferative window [75,76]. An excess amount of fatty acids was also reported to induce cell death in H9C2 cardiomyoblasts (via a ferroptosis mechanism) [54,77] or endothelial dysfunction (via increased oxidative stress, inflammation, mitochondrial dysfunction and apoptosis) [78]. Therefore, early changes in circulating lipids or other metabolic substrate levels observed in PNOF animals may directly impact cardiomyocytes’ ability to proliferate in the immediate postnatal period and could have a long-term impact on cardiac hypertrophy and myocardial susceptibility to pathological stressors.

Finally, histological analysis with picrosirius red showed that adult male overfed mice have an increased collagen deposition in the left ventricle in non-pathological conditions [32,37,79]. Adult overfed rats also present higher interstitial and perivascular collagen density [29], and this higher fibrosis content is combined with greater expression and activity of the matrix metalloproteinase-2 (MMP-2) [6]. Whether this reflects a direct effect of PNOF on cardiac fibroblasts remains to be established. However, such ventricular structural changes linked to higher collagen deposition might be responsible for modifications of the ventricular architecture and the alteration of contractile function [29]. Moreover, an increase in the mRNA expression of atrial natriuretic peptide (ANP) and brain natriuretic peptide (BNP) in the hearts of PNOF mice strongly suggests the activation of pro-hypertrophic pathways [37].

### 4.2. PNOF Induces Short- and Long-Term Alterations of Gene Expression in the Heart

In order to explain the long-term metabolic and cardiovascular alterations induced by neonatal overnutrition, foetal programming through gene expression modifications has been suggested. It has already been observed that obesity during pregnancy increases the risk for the offspring to develop cardiovascular diseases in humans and animals [80]. The expression alteration of crucial genes was reported, such as upregulation of Myh7, coding for the β-myosin heavy chain (β-MHC), brain natriuretic peptide (BNP) or Gata-4 during foetal development, potentially leading to increased cardiovascular risk. Similarly, a comparison of gene expression between the hearts of over and normally fed mice at weaning showed that the expression of not less than 822 genes was modified after PNOF [32]. After deeper analyses, results were restricted to 102 over- or under-expressed genes, and among them, 12% of the over-expressed genes were structural encoding genes mostly for collagen.

Gene expression is also regulated by epigenetic modifications through histone acetylation, DNA methylation or RNA-based mechanisms, and early postnatal nutrition may program obesity and cardiac risk via epigenetic mechanisms [81]. In infants, the duration of breastfeeding has an impact on metabolic epigenome [82]. In PNOF rats, Plagemann was the first to report an increased hypothalamic methylation in the promoter of the anorexigenic proopiomelanocortin gene [53], demonstrating epigenetic mal-programming of brain satiety pathways. Other groups have described epigenetic changes in skeletal muscle [83], the liver [84] or the pancreas [85] associated with insulin resistance in PNOF. In the heart, a study analysed the impact of maternal exposure to high fat diet on epigenetic markers of the offspring in rats. They reported alterations of DNA and histone methylation leading to the activation of pro-hypertrophic and pro-fibrotic genes in the heart, which might have a role in the PNOF-induced cardiovascular risk increase [86]. Moreover, an alteration of the NLRP3 inflammasome pathway in the myocardium was noted [33]. Its signalling is regulated by the micro-RNA miR-193b, which is also impaired by PNOF. The involvement of altered micro-RNA regulation has been evidenced in the cardiac health of offspring exposed to maternal high-fat diet [87], and this could play an important role in the deleterious effects of PNOF.

### 4.3. PNOF is Associated with Increased Oxidative Stress and Mitochondrial Impairment in the Heart

Mitochondria, organelles that are particularly abundant in cardiac cells, play a central role in cardiomyocyte function and in the production of reactive oxygen species. Therefore, mitochondrial dysfunction may lead to excessive free radical production and to the impairment of ventricular bioenergetics. In the post-weaning hearts of PNOF mice, ultrastructural analysis demonstrated mild mitochondrial damage without alterations in oxidative phosphorylation complexes [38]. A study was conducted to explore mitochondrial function under physiological or stressful conditions such as anoxia/reoxygenation (A-R) in the hearts of rats subjected to PNOF [25]. They observed a dysregulation of mitochondrial respiratory capacity in young and adult animals, especially after stimulation with ADP (state 3). Although A-R induced a decrease in respiratory parameters in mitochondrial cardiomyocytes, it did not worsen mitochondrial function in overfed rats in comparison to control rats.

In cardiomyocytes, reactive oxygen species (ROS) are mainly produced by complexes I and III of the respiratory chain, thus, mitochondrial dysfunction may lead to an increased production of ROS. An imbalance between ROS production and the activity of antioxidant enzymes, such as superoxide dismutase, catalase and glutathione peroxidase, may induce oxidative stress and has been described in adult overfed hearts [6,11,29,32]. The direct evaluation of the production of reactive oxygen and nitrogen species with electron paramagnetic resonance spectroscopy [88] has demonstrated increased production of these species in the hearts of PNOF rats and mice [12,32]. Additionally, an increase in markers of oxidative lipid damage, 4-hydroxynonenal, has been observed in juvenile PNOF mice hearts [38]. Oxidative stress has been shown to activate hypertrophic and pro-inflammatory pathways and thus could be, in part, responsible for cardiac hypertrophy and higher collagen deposition, as previously mentioned [89]. Indeed, inflammation and immune cells are crucial in the initiation, development, and progression to chronicity of several cardiovascular diseases, such as hypertension [90], atherosclerosis [91] or pathologies affecting cardiac injury and repair [92] and involve the innate and adaptive immune responses [93,94]. Again, these biochemical changes associated with higher inflammatory status and oxidative stress might be involved in the alterations of cardiac contractile function and predispose the heart to a higher sensitivity to ischemic or toxic injuries.

### 4.4. PNOF Alters Cardiac Function In Vivo

Echocardiographic analysis enables us to study how PNOF affects the global cardiac function in vivo in young and adult overfed animals. Interestingly, 3-month-old overfed male mice initially display a significantly improved left ventricular ejection fraction (LVEF) after PNOF in comparison to control mice. However, it gradually decreases over time, resulting in a significant reduction in LVEF at 7 months, which is associated with an increased left ventricular internal diameter [12,32]. Another study in rats demonstrated a significant increase in left ventricular posterior wall and interventricular septum thickness at 120-days-old, but no differences in ventricular internal diameter or contractile function [29]. The impact of an obesogenic maternal diet and/or a post-weaning obesogenic diet on cardiac function was evaluated in 8-week-old male mice. In either case, a decrease in LVEF and in left ventricular fractional shortening (LVFS), associated with an increase in systolic blood pressure and heart fibrosis, was observed. Both diets induced the alteration of cardiac contractility, but only maternal diet led to a rise of the Myh7/Myh6 ratio, which represents an indicator of cardiac hypertrophy. Moreover, dietary fat exposure during gestation and lactation predisposed the offspring to develop cardiac hypertrophy, ROS accumulation, apoptosis, fibrosis and cardiovascular diseases in adult life [95]. These results suggest that perinatal nutritional programming mechanisms leading to cardiac dysfunction might be distinct in the pre and postnatal period. However, both models support the concept that perinatal periods are of major importance to determine young- and adult-onset obesity and subsequent cardiovascular risk.

Surprisingly, cardiac alterations were shown to be gender-dependant. Litter size reduction does not affect the cardiovascular function of Wistar female rats at 150 days of life as it is described for males, who present myocardial hypertrophy and left ventricle structural remodelling [31]. Oestrogen might act directly on cardiac myocytes by increasing atrial natriuretic peptide, which may decrease apoptosis/necrosis and lead to mitigating the cardiac hypertrophy [55]. However, more experimental data are needed considering the lack of information regarding PNOF-induced cardiac alterations in female rodents.

### 4.5. PNOF Increases Myocardial Susceptibility to Pathological Stresses: Ischemia-Reperfusion Injury and Cardiotoxic Drugs

Since structural cardiac and vascular dysfunctions were described in physiological conditions, additional studies have explored the cardiovascular response to pathological situations such as ischemia-reperfusion (I-R). Again, an important variability in the findings between studies was observed. Before the induction of ischemia in hearts isolated and perfused in basal conditions, some authors reported that hearts from PNOF rodents had a decreased cardiac contractility [37], while we never observed such initial alterations of left ventricular function neither in PNOF rats nor in mice [6,12,32]. After 30 min of ischemia, an impairment of myocardial contractility, characterized by a decrease in dP/dt and left ventricular developed pressure, was reported in overfed hearts [6,20,37] in comparison to their control littermates. Cardiac output recovery was also significantly lower in overfed animals and associated with the increased release of lactate dehydrogenase, a marker of cell injury [6]. An increase in an apoptotic marker, caspase 8, in response to PNOF and I-R was also observed [20]. The alteration of important signalling pathways was also reported in overfed ischemic hearts, such as the phosphorylation activation of STAT-3, one component of the SAFE pathway, which is known to be protective against I-R injury, or the overactivation of SOCS-3, a potential inhibitor of STAT-3 activation [32,44]. Significant alterations in heart metabolism proteins, including Akt2, pAkt/Akt1, pAkt/Akt2, AMP-activated protein kinase (AMPK), pAMPK/AMPK, protein-tyrosine phosphatase 1B (PTP1B), IRS1, fatty-acid-binding proteins (FABP) and cluster differentiation 36 (CD36) were also reported in PNOF mice hearts after ex vivo ischemia-reperfusion injury [37].

Impaired recovery after ex vivo I-R was associated with an increased infarct size in the overfed group in comparison to its control [12,25,32], as well as an increased collagen deposition [37]. Recent data from our teams also demonstrate a long lasting (up to 12 months after PNOF) increased sensitivity to myocardial ischemia-reperfusion injury induced by in vivo transient ligation of the left anterior descending (LAD) coronary artery in male mice (unpublished data [34]). Once again, males and females did not present the same cardiac sensitivity to I-R, with PNOF females being less affected by myocardial ischemia-reperfusion injury (unpublished data). More investigations are therefore needed to explain the mechanisms potentially involved in the alteration of cardioprotective pathways and in sexual dimorphism in PNOF mice.

Some anticancer drugs, such as anthracycline doxorubicin (DOX) and specific antibodies like trastuzumab, are known to induce dose dependent toxicities, which sometimes act in a synergic deleterious manner [96]. DOX is well known to be involved in the alteration of cardiac gene expression [97], leading to cardiomyocyte death and impaired cardiac function especially in obese animals [98] or in overweight or obese cancer patients [99]. PNOF mice treated with sub-lethal mild toxic doses of DOX showed enhanced impairment of left ventricular systolic function than control littermates, whereas the expression of β-MHC increased significantly in cardiac tissue [100]. These results demonstrate that adult PNOF mice were more sensitive to cardiac systolic impairment due to DOX cardiotoxicity.

Altogether, these findings suggest a greater sensitivity of PNOF hearts to stressful pathological conditions associated with oxidative stress, such as ischemia-reperfusion injury or exposition to cardiotoxic anticancer drugs.

## 5. Openings

As developed in this review, PNOF negatively impacts cardiovascular function. Other nutritionally challenging situations, such as peri- and/or postnatal undernutrition, may also have deleterious effects on the heart’s structure and functioning [101,102,103]. The alteration of some parameters persists over time and can even worsen. Nevertheless, some of the functional consequences of PNOF can be prevented or reversed. In adulthood, one month of 20% food restriction in adult male mice improved insulin plasma level, ischemia-reperfusion sensitivity and cardiac function and decreased oxidative stress [12]. This calorie restriction was also able to reduce PNOF-induced liver senescence [104] and other hepatic disorders such as steatosis and oxidative stress [105]. Furthermore, the early administration of metformin, a drug commonly used as a type 2 diabetes treatment, prevented PNOF-induced metabolic disorders such as hyperinsulinemia and improved pancreatic function in postnatal overfed rats [106]. Another study also demonstrated that a training period of 8 weeks was beneficial for improving the cardiorespiratory function represented by VO_2_ max as well as glucose and insulin sensitivity in rats subjected to PNOF, regardless of the training intensity [26].

Finally, it is noteworthy that, in this review, we mainly focused on the PNOF cardiovascular effects on male offspring. As briefly mentioned, PNOF may affect female rodents in a very different manner [31], and cardiometabolic disorders are sex-dependent in several aspects [107]; therefore, more data would be necessary to determine the distinct PNOF-induced consequences on both genders.

## 6. Conclusions

Postnatal overfeeding by litter size reduction is associated with numerous metabolic and hormonal alterations, which may lead to long-term deleterious cardiovascular consequences, such as arterial blood pressure elevation, ventricular remodelling, cardiac dysfunction and increased sensibility to ischemia-reperfusion. Indeed, litter size strongly influences adult metabolism and organ function in adults [108], and scientific articles that utilize rodent models for obesity and metabolic research should include more detailed information on the litter sizes. The results may somehow differ by number of pups per dam, strain type, diet composition or age of animals during the experiments; however, they all confirm that the nutritional state in the immediate postnatal period is crucial for proper myocardial and vascular structuration, metabolism fate and appropriate response to pathological conditions.

## Figures and Tables

**Table 1:** Summary of early (**[E]**: birth to 1-month-old) and long-term (**[L]**: 1 to 12-months-old) cardiovascular and metabolic alterations induced by the postnatal overfeeding of male rodents. ↑: significantly increased compared to control; ↓: significantly decreased compared to control; =: similar to control.

SPECIES: Strain	[E]: Early Alterations (Birth to 1-Month-Old) or [L]: Long-Term Alterations (1 to 12-Months-Old)	Ref.
RATS: Sprague Dawley	**[L]**: ↑ blood pressure Glomerulosclerosis Renal dysfunction	[18]
RATS: Sprague Dawley	**[L]**: ↑ blood pressure Renal cell apoptosis and inflammation	[19]
RATS: Sprague Dawley	**[L]**: ↑ plasma leptin levels ↑ susceptibility to ex vivo ischemia-reperfusion ↑ apoptotic markers	[20]
RATS: Sprague Dawley	**[E]**: ↑ plasma glucose/insulin/leptin/total lipids/cholesterol levels ↓ HDL cholesterol ↓ vasodilation to acetylcholine, SNP and insulin Cardiac and vascular insulin resistance Pro-inflammatory and pro oxidant state	[21]
RATS: Wistar	**[E]**: ↑ plasma glucose/insulin/leptin levels Alteration of cardiac insulin and leptin pathways	[22]
RATS: Wistar	**[E]**: ↑ plasma total cholesterol/triglycerides levels Left ventricular hypertrophy ↓ intramyocardial vessel density	[23]
RATS: Wistar	**[L]**: ↑ plasma insulin/leptin levels = glucose levels Glucose intolerance ↑ glucose-stimulated insulin secretion GLUT2 ↑ insulin content in pancreatic islets	[7]
RATS: Wistar	**[L]**: ↑ blood glucose/plasma insulin/leptin levels = triglycerides/cholesterol levels = vasoreactivity to acetylcholine/SNP/phenylephrine ↑ collagen deposit ↑ susceptibility to ex vivo ischemia-reperfusion Alteration of oxidative balance	[6]
RATS: Wistar	**[L]**: ↑ blood pressure ↑ aortic thickness ↓ aortic elastin integrity ↑ aortic MMP-2 expression	[24]
RATS: Wistar	**[E]**: ↑ plasma glucose/cholesterol/triglycerides levels Alteration of mitochondrial function Alteration of oxidative balance **[L]**: ↑ plasma glucose/triglycerides levels Alteration of mitochondrial function Alteration of oxidative balance	[25]
RATS: Wistar	**[L]**: ↑ blood glucose Glucose and insulin intolerance	[26]
RATS: Wistar	**[L]**: ↑ plasma insulin/triglycerides levels = glucose/HDL and LDL cholesterol levels ↑ blood pressure ↑ aortic collagen content ↓ elastic fiber density	[27]
RATS: Wistar	**[E]**: ↑ glomerular size and cellularity ↑ renal collagen content Alteration of oxidative balance	[28]
RATS: Wistar	**[L]**: ↑ plasma triglycerides ↓ HDL cholesterol ↓ vasodilation to acetylcholine ↑ vasoconstriction to phenylephrine ↑ blood pressure Cardiac hypertrophy and fibrosis = cardiac contractile function Alteration of oxidative balance	[29]
RATS: Wistar	**[L]**: ↑ blood pressure Cardiomyocyte hypertrophy Cardiac fibrosis	[30]
RATS: Wistar	**[L]**: ↑ blood pressure = cardiac contractile function Cardiac hypertrophy	[31]
MICE: C57BL/6	**[E]**: Alteration of cardiac gene expression **[L]**: ↑ plasma cholesterol/insulin/leptin levels ↑ blood pressure ↓ cardiac contractile function ↑ collagen deposit ↑ susceptibility to ex vivo ischemia-reperfusion Alteration of myocardial oxidative balance	[32]
MICE: C57BL/6	**[L]**: ↑ plasma insulin/leptin levels = glucose levels Glucose and insulin intolerance ↓ cardiac contractile function ↑ collagen deposit ↑ susceptibility to ex vivo ischemia-reperfusion ↑ cardiac oxidative stress	[12]
MICE: C57BL/6	**[L]**: ↑ plasma IL-6 levels Alteration of cardiac inflammasome NLRP3 and insulin pathways	[33]
MICE: C57BL/6	**[L]**: ↑ infarct size after in vivo ischemia-reperfusion ↓ cardioprotective pathways	Unpublished data [34]
MICE: C57BL/6	**[E]**: ↑ senescence pathways in kidney **[L]**: ↑ blood pressure ↑ glomerular number	[35]
MICE: Swiss	**[L]**: ↑ plasma insulin levels = glucose/triglycerides/cholesterol levels Glucose intolerance Alteration of cardiac insulin pathway ↑ heart weight	[36]
MICE: Swiss	**[L]**: ↑ plasma glucose/insulin/leptin levels ↑ collagen deposit Cardiac hypertrophy ↑ susceptibility to ex vivo ischemia-reperfusion Alteration of insulin and fatty acid pathways	[37]
MICE: Swiss	**[E]**: ↑ plasma glucose/insulin/triglycerides/total cholesterol levels Alteration of cardiac insulin pathways Cardiac hypertrophy and fibrosis Alteration of cardiac metabolism	[38]
